# 

**Published:** 1889-06

**Authors:** 


CONTENTS.

Vol. 36 No. 6.

| | PAGE. |
|---|---|
| Special | 121 |
| Decoration Day | 121 |
| Health and Hell | 122 |
| Feeding and Nursing the Sick | 126 |
| Vaccination—Its uses and abuses | 130 |
| The Ivory Plant | 133 |
| Nirvana | 135 |
| The Remedies of Nature | 136 |
| The Religion of Buddha | 138 |
| Eccentricities in Diet | 139 |
| Want of Sleep | 140 |
| Book Notices | 141 |
| Household | 142 |
| Miscellaneous | 143 |
| Only, O Man | 144 |

INDEX TO ADVERTISEMENTS OF HALF A PAGE AND OVER.

| | PAGE. |
|---|---|
| Murdock's Liquid Food Co | I |
| Esoteric Publishing Co | II |
| Celluloid | II |
| Hollow Suppositories | III |
| E. C. Morris & Co | IV |
| Lactopeptine | V |
| Revised Premium List | VII |
| The Herb Cure Co | IX |
| Ladies' Home Companion | X |

